# Women’s sport and everyday resistance

**DOI:** 10.3389/fspor.2023.1007033

**Published:** 2023-07-26

**Authors:** Risa F. Isard, E. Nicole Melton, Charles D. T. Macaulay

**Affiliations:** Sport Management Program, Department of Educational Leadership, Neag School of Education, University of Connecticut, Storrs, CT, United States

**Keywords:** everyday resistance, women’s sport, intersectionality, women athletes, social justice, sport in society, sport and social change

## Abstract

This paper presents a conceptual model to understand the relationship between everyday resistance and women’s sport. Everyday resistance refers to when members of an oppressed group engage in mundane actions (i.e., playing sports) to resist dominant power structures and social norms. After reviewing resistance literature, we identify two levels of everyday resistance for women’s sport*: women’s sport as everyday resistance* and *everyday resistance within women’s sport*. The former refers to when women participate in sport, thereby challenging social norms that marginalize women in society and exclude them from sport. The latter refers to how women athletes with intersecting marginalized identities resist the norms of who participates in women’s sport and how, given the norms of sport that privilege whiteness, heteronormativity, and higher social classes among others. The model we introduce advances both sport scholarship and everyday resistance literature and can help scholars conceptualize how women create change in sport and in society—as well as how women athletes create change within women’s sport, specifically.

## Introduction

*“We are a walking protest at all times as a W.N.B.A. athlete.”*

- Mistie Bass, retired WNBA player and champion

*“Women using their bodies purely for their own power and pleasure is still a revolutionary act.”*

- Lauren Fleshman, two-time 5,000 m US champion

The quotes by Bass and Fleshman illustrate how some contemporary women athletes perceive their pursuit in sport. Despite their differences in sport and life—Fleshman is a white, openly bisexual individual sport athlete and Bass is a Black team sport athlete—they share an understanding that women’s sport has a social meaning (1, 2) such that simply being a woman athlete makes a statement about women’s role in society and sport. Their words point to typically unnoticed and seemingly mundane actions—playing sports—women undertake that influence both sport and the broader culture. That is, they articulate an understanding of the *everyday resistance* of women athletes. In this conceptual paper, we explore what makes women’s sport an act of everyday resistance as well as how such everyday resistance manifests.

Everyday resistance (3)—when members of an oppressed group engage in mundane actions to resist dominant power structures and social norms—can help explain the social significance of women’s sport. Scholars have demonstrated that women often use acts of everyday resistance to affect change and advance gender equality. For example, women embody everyday resistances through taking pride in fat bodies (resisting Westernized beauty standards that emphasize thinness for women) and embracing personal aesthetics such as hairiness [resisting Westernized beauty standards that suggest hair is unclean and unfeminine (4)]. In this way, everyday resistance among women is less about outright activism (i.e., marches, boycotts, walkouts) and more about embracing the feminist ethos that “the personal is political.” Simply, the way a woman shows up in her everyday life has implications.

While resistance scholars have studied the everyday resistance of women, less is known about the use of sport in this manner. Indeed, sport is an embodied exercise that everyday resistance scholars have largely overlooked. The limited resistance scholarship that has addressed sport has tended to center race and ethnicity as axes of marginalization, at times with an explicit focus on men (5–7). These studies demonstrate how everyday resistance can help scholars understand the role of sport. However, they do not specifically consider the experiences of women, which may be unique given the gendered context of sport.

Thus, the purpose of this conceptual paper is to articulate a model for understanding everyday resistance and women’s sport. We focus on the United States. Our conceptualization extends the framework of everyday resistance of Johansson and Vinthagen (4). Specifically, we articulate two levels for understanding the relationship between everyday resistance and women’s sport: *women’s sport as everyday resistance* and *everyday resistance within women’s sport*. In doing so, we contribute to both sport and resistance scholarship. For the former, we further develop the idea of everyday resistance [c.f. (6)]. For the latter, we further advance the conceptual understanding of the role of sport in everyday resistance. As we explicitly center women’s gender and take an intersectional approach, we further the conceptual knowledge in both bodies of literature (4–7).

Below we present a conceptual model for understanding women’s sport and everyday resistance. We start by reviewing resistance literature. Next, we define two levels of everyday resistance—women’s sport as everyday resistance and everyday resistance within women’s sport—and offer examples for each. Finally, we suggest how scholars could use this model as well as note opportunities to further development.

## Resistance literature

Below we review relevant literature on resistance. We emphasize the components of resistance and its relationship to gender and sport, before turning our focus to everyday resistance, specifically.

### Resistance

While there is no one agreed upon definition of resistance, scholars generally agree that resistance includes two characteristics: action and opposition (8). First, a requisite for action means that resistance involves some behavioral element, whether verbal, cognitive, or physical. Second, opposition refers to how resistance takes a stand against someone or something (8).

Beyond finding consensus around action and opposition, scholars actively disagree on the importance of two other commonly discussed characteristics of resistance: intention and recognition. Intention refers to the state of mind of the resister; recognition refers to whether the target of the action or onlookers understand the act as resistant (8). While scholars disagree on whether intention is necessary—as well as whether others must understand an act as resistance—actions that lack both intention and recognition are not considered resistance at all (8).

### Resistance and sport

Sport scholars have long sought to understand the potential of sport to create social change. This question about if and how sport can serve as a site of resistance is situated within a conversation about how sport at times perpetuates inequality and oppression, including in ways that disadvantage women (9). Despite these dynamics, athletes have undertaken activist efforts, drawing scholarly attention [e.g. (9–11)]. Such activism involves actions that are adjacent to, but ultimately separate from, participating in the sport itself, such as fist-raising, kneeling, and custom-designed warm-up shirts. As these actions are (by design) highly visible and contentious, those who engage in these practices often face strong backlash (9).

While backlash to as well as research on activism in sport demonstrates the impact athletes can have, grand actions are not the only way to use sport for social progress (12). Indeed, at times, the seemingly mundane action of simply participating in sport itself can represent resistance and engender social change. Scholars who have taken this approach have used a variety of theoretical perspectives. These include the following: hegemony (13–16); queer and feminist theories [e.g. (14, 17–21)]; and Foucauldian approaches (16, 22, 23). Some of this research has specifically sought to address how sport is a potential space of resistance for women (19–23). While these studies indirectly address concepts related to everyday resistance—such as how women challenge the status quo in or through sport—there has been a notable dearth of research that has conceptualized everyday resistance as Scott theorized. However, everyday resistance provides a lens to help scholars glean additional insights and be precise in articulating who is resisting, what they are resisting, and how they are resisting.

### Everyday resistance

Traditionally, scholars used ideas about resistance to study mass movements (8, 24). However, such a focus on large-scale actions and open revolutions overlooked smaller-scale, everyday actions that resist power structures (3). To draw attention to these means of resistance, Scott (3) coined the term everyday resistance. For Scott, everyday resistance is characterized by small-scale acts with cumulative effects, concealment, and a focus on de facto changes. That is, everyday resistance focuses on the additive impact that many small acts have on power structures or social norms [see also (23)]. Second, everyday resistance is relatively concealed, often embracing actions that those with power may not readily recognize as resistance but nevertheless have meaning and potential to shift social relations. Third, everyday resistance focuses on de facto shifts such that the focus is on loosening social norms rather than changing policy (24).

Limited research has adopted everyday resistance as Scott and others have articulated to understand power and protest within sport. Two notable exceptions focus on the role of basketball in contesting racial and ethnic identities (5, 6). These studies identify how basketball can contribute to resistance through direct competition (e.g., a Lakota high school team playing against a White team) (5), instigating temporary shifts in relations. Separately, a historic interpretation of the sport activities of the Aboriginal individuals suggests that sport provided opportunities for resistance, defiance, and opposition, even within the confines of Westernized practices (7). We build on these understandings by both centering women’s gender in our paper, taking an intersectional approach to the experiences of women, and demonstrating the applicability of everyday resistance across sporting contexts (i.e., running, baseball, gymnastics, ice skating). In addition, we focus on the role of sport in creating longer-lasting changes in cultural norms that permeate sport settings as well as broader society.

#### Dimensions of everyday resistance

A recent conceptualization of everyday resistance offers a framework for scholars. Johansson and Vinthagen (4) identify the key components of resistance that effectively answer the questions: who is resisting against whom/what, where, when, and how? The answers to these questions represent the four dimensions of everyday resistance of Johansson and Vinthagen (4): repertoires, relationships of agents, spatialization, and temporalization. We discuss each briefly.

First, repertoires are the “how,” defined as “a collection of ways or methods of resistance that people are familiar with, know of, understand and are able to handle” [(4), p. 422]. For example, the United States Women’s National Soccer Team (USWNT) has exemplified everyday resistance since its debut in 1985. Putting aside the team’s historic acts of outright protest and labor negotiations that sought legal and policy remedies, the team’s playing of soccer—a sport traditionally the domain of men (26)—at the highest level represents a social significance that makes it ripe for understanding it through a lens of everyday resistance. Notably, the 1999 team (“the 99ers”) showcased “women who were strong and driven and determined and powerful” [(27), para. 5, quoting Scurry]. They did this through playing soccer, a repertoire that represented the tool at their collective disposal as elite athletes.

Second, the relationships of agents refers to the “who, against whom/what.” Understanding actors and how they relate to one another is critical to understanding the meaning of actions. There are three known agent types: resisters, targets, and observers (4, 8). First, resisters are those who are doing the resistance. Second, targets—who or what at which the resistance is aimed—may be individuals, groups, organizations, institutions, and social structures (8). Lastly, observers are “onlookers” (8) to the resistance. While observers are often overlooked in the literature (4), they are critical to assessing the meaning that others make of actions, known as “the social understanding” of the resistance (28), p. xi. In the example of the 1999 USWNT, the players collectively and individually were resisters. Their targets were the historic male domain of sport as well as broader societal gender norms. Lastly, live spectators, those watching on television, and individuals following through media were all observers. As witnesses to the 99ers, the audience members interpreted and constructed a meaning of significance (29). Thus, regardless of whether the UWSNT had a conscientious intention to engage in resistance, the meaning that others made from the USWNT set them up to be resisters (26).

Third, spatialization goes beyond a literal and narrow understanding of “where” to recognize that space is socially constructed (4). Where resistance happens is important because social life is structured in that site in a specific way. For example, the location of the 1999 Women’s World Cup—the United States—informed the resistance of the 99ers. Despite the progress of the United States over the years compared with other countries, women athletes were still treated as afterthoughts—signified by limited media coverage, unequal pay, and disparities in travel conditions, marketing, and playing surfaces (30). Thus, spatialization provides context for resistance.

Fourth, temporalization goes beyond the “when” to include an understanding that time is also socially constructed (4). The 99ers did not just show up to play in a World Cup, but devoted years to the pursuit during a time when elite women’s sport was hardly seen as legitimate (30). Temporalization and spatialization are inseparable (4), as all actions occur in a specific time and space that are mutually and socially constructed.

## Context of women’s sport

The present conceptual model focuses on women’s sport, by which we mean sporting activities in which women participate and compete. We recognize that women participate in a variety of sports, each with their own characteristics and contexts. Notably, some sports may be gender-typed as more feminine and thus more “appropriate” for women, while other sports may be perceived to be more masculine and thus less appropriate for women (31–33). Though some sports may be deemed more appropriate for women (32), sport still largely remains the domain of boys and men (34, 35). Given this continued privileging of men’s sport and devaluing of women’s sport, we suggest that everyday resistance is available through all forms of women’s sport; however, how that resistance manifests may differ (a conceptualization of these differences is outside the scope of the initial model presented here). Likewise, though the conceptual model we build below employs examples from (adult) women’s sport, we contend that similar patterns likely appear in girls’ youth sport, too.

### Theoretical assumptions

Our adoption of everyday resistance to understand women’s sport relies on four assumptions. First, we assume that sport holds a particular status that makes people pay attention to it. Second, we assume that sport affords athletes a particular kind of status (36). We believe this is true for athletes at all levels, though we recognize that some athletes have much greater status than other athletes. Athletes who are men may, on average, have greater status than athletes of other genders (37). Likewise, athletes without disabilities may, on average, have greater status than athletes with disabilities (38). But even athletes with low status relative to other athletes have more status than a non-athlete who is not otherwise a celebrity. Because of the cultural status of sport and the attention paid to athletes, observers hold a particularly important role for sporting everyday resistance in all levels described below. Observers may include other women athletes, people of other genders, and passersby and enthusiastic spectators alike. Third, we assume that if someone is involved with sport, they have likely been exposed to sport since a young age. Through early exposure, individuals of all genders and backgrounds are socialized to the norms and expectations of sport (39). Fourth, we focus on women, defined broadly and inclusively. Other genders (i.e., men and non-binary individuals) likely resist in and through sport; their exclusion from this paper should not imply that they do not. Rather, given the importance of spatialization and temporalization, athletes of other genders may perform everyday resistance differently. Those resistances are worthy of their own exploration and understanding.

## Manifestations of everyday resistance and women’s sport

We assert that there are two levels of everyday resistance in relationship to women’s sport: women’s sport as everyday resistance and everyday resistance within women’s sport. Below we define these levels and illustrate their characteristics through examples from past and recent history. See Table 1.

**Table 1:** Everyday resistance and women’s sport.

Dimension^a^	Women’s sport as everyday resistance	Everyday resistance within women’s sport
**Definition**	Women using sport to resist societal gender norms, including (but not limited to) women’s exclusion from sport	Women in sport challenging who is allowed to participate in women’s sport and how, using their own participation as an act of resistance
**Repertoire**	Participating in a sport	Participating in a sport
**Relationship of agents**
** Actor**	A woman	A woman with intersecting marginalized identities
**Target**	Societal norms that subjugate women	Norms of women’s sport that exclude and marginalize women with intersecting marginalized identities
	Norms of sport that exclude and marginalize women	
** Observer**	Spectators, other athletes, media, any witness	Spectators, other athletes, media, any witness
**Spatialization**	Norms that dictate sport as the domain of men	Norms that dictate women’s sport as for women with privilege (class, racial/ethnic, sexual orientation, etc.)
**Temporalization**	Norms that dictate appropriate ways for women to spend their time	Norms that disparately dictate appropriate ways for marginalized women to spend their time
**Examples**	Justine Siegal Bobbi Gibb	Tiffany Chin Nia Dennis WNBA players

^a^ Repertoire, relationship of agents, spatialization, and temporalization are identified by Johansson and Vinthagen (4) as the key dimensions for understanding everyday resistance.

### Women’s sport as everyday resistance

Women’s sport as everyday resistance means that by merely engaging in sport—by sporting while woman—women are performing everyday resistance. In this way, sport is a repertoire of resistance for women. Using the framework of the four dimensions of everyday resistance of Johansson and Vinthagen (4), we understand that this level of everyday resistance is the result of spatial arrangements that have marked sport as a space for men since the origins of modern sport (6, 13). Further, temporal norms inform this level. Temporal norms dictate appropriate ways for women to spend their time. For example, socially enforced ideas emphasize women’s fertility (a time-bound phase in a woman’s life) as a reason they should not engage in sport (37). Lastly, we note that in this level, resisters are women athletes who simultaneously target social arrangements that are limiting women across society and the norms excluding women from sport.

To understand women’s sport as everyday resistance, consider Justine Siegal. Siegal grew up playing baseball and receiving messages that girls did not belong in the sport. The more coaches and others discouraged her from pursuing the sport, the more she devoted herself to it. She eventually made history as the first woman to throw batting practice for a Major League Baseball (MLB) team. Siegal uses sport to resist societal messages about what is appropriate for girls, in society broadly and in sport specifically. As Siegal is known to ask, “If you tell a girl she can’t play baseball, what else will she think she can’t do?” [(38), para. 13, quoting Siegal]. For this reason, Siegal believes playing baseball is “a social justice issue, and that is why I am so passionate about it” [(38), para. 13, quoting Siegal]. This is women’s sport as everyday resistance: a woman using sport to resist societal gender norms and exclusion from sport.

Running pioneer Bobbi Gibb likewise represents women’s sport as everyday resistance. Gibb became the first woman to run the Boston Marathon when she disguised herself as a young man to complete the 26.2-mi course in 1966 (42). The Boston Marathon is the flagship event of the Boston Athletic Association (BAA), which boasted a founding mission “to encourage all *manly* sports and promote physical culture” [(43), para. 1; emphasis added]. Accordingly, Gibb’s goal was to partake in the massive spectacle “to make people see something different that would shake them up a little bit, maybe change some traditional attitudes” [(44), para. 2, quoting Gibb]. After her official entry was rejected because of her gender, she knew her race in disguise would be more than simply a personal achievement (42). She thought, “if I failed to finish, I would set women back another 50 years—maybe more” [(42), para. 34, quoting Gibb]. Gibb’s run was an act of everyday resistance as she used the marathon to oppose views about the place of women in society and the exclusion of women from the historic Boston race.

Women’s sport represents everyday resistance when women participate in sport in a manner that challenges social norms for where and how women should exist in society broadly as well as the norms that exclude women from sport. Uniquely, women’s sport may be particularly effective as a tool of resistance both when there are few women participating and when participation is more widespread. For the former, consider the statement Gibb and Siegal made as the first women to reach their achievements. The messages of their sports participation were in part possible because other women were not participating. However, as more women participate in sport, their collective impact accumulates, challenging the fabric of oppressive norms of society and sport in an exponential manner.

### Everyday resistance within women’s sport

Everyday resistance within women’s sport refers to the way that women athletes resist the norms of who participates in women’s sport and how. It recognizes the norms that govern the sport privilege socio-historic traditions of women such as whiteness, heteronormativity, and class-privilege (45). Thus, specifically for women with intersecting marginalized identities, participating in sport is a repertoire of resistance. Using the framework of the four dimensions of everyday resistance of Johansson and Vinthagen (4), we understand that everyday resistance within women’s sport is the result of spatial arrangements that have marked women’s sport as a space for certain types of women (45). Further, temporal norms inform this level, as women with intersecting identities navigate socially prescribed ideas about appropriate ways specifically for class-, race-, or otherwise-marginalized women to spend their time. That is, even when women are allowed to participate, that privilege may only extend to certain women (i.e., white, class-privileged, straight). As such, we note that in this level, resisters are women athletes with intersecting identities who target social arrangements that exclude marginalized women from sport.

To understand everyday resistance within women’s sport, consider Tiffany Chin, the first Asian-American athlete to represent the United States in any sport at a world championships or Olympics (46). Chin stood out from other women figure skaters. She was the lone athlete of color to compete in the 1982 Ladies’ Figure Skating US Championships (47). Comments calling attention to Chin’s race came from peers and television personalities alike. When she was young, one athlete peer told her, “You’re really good, but you know you’ll never be a champion. Figure skating champions have blonde hair and blue eyes, and you don’t have either” (47). After making a name for herself, she had to contend with the pejorative nickname “China doll” (46). Given the standards of white femininity applied to women figure skaters (48), Chin was engaged in everyday resistance within women’s sport, contesting the norms about who was allowed to participate.

Gymnast Nia Dennis also embodies everyday resistance within women’s sport. The 2021 college floor routine of Dennis went viral as she performed to an all-Black mix of musical artists while incorporating symbols of Black activism and Black Greek organizations. Given the history of women’s gymnastics as a largely white sport (49), Dennis’s presence as a Black athlete—and her explicit celebration of Black culture through her floor routine—represents everyday resistance within women’s sport. Indeed, Dennis was among the few Black gymnasts in her hometown gym, where she faced frequent comments about her hair and body (50). Moreover, her elite gymnastics training represented the sport’s White norm of being “cookie-cutter, very ballet, very classical” [(50), para. 27, quoting Dennis]. Dennis seized the opportunity to show her full personality and self in college gymnastics, challenging the status quo by performing in a way that is “modern, new, urban” [(50), para. 27, quoting Dennis]. This—challenging who is allowed to participate in women’s sport and how—is everyday resistance within women’s sport.

As one final example, we consider how Women's National Basketball Association (WNBA) players at present challenge the norms of women's sport. Women’s sport has long asked women athletes to present in a hyper-feminine manner—a kind of compromise or accommodation between women athletes and the male preserve of sport (51). WNBA players have resisted this norm in recent years, embracing androgynous and masculine couture. Notably, the gap has narrowed between how a WNBA athlete expresses themselves off the court and in play (51, 52). Today “There is more than one way to be a woman in basketball,” women’s sport journalist de la Cretaz concluded [(51), para. 15]. Embracing androgynous or masculine gender presentations resists the historic norms of women’s sport, illustrating everyday resistance within women’s sport.

Everyday resistance within women’s sport relies upon athletes with intersecting marginalized identities staking out space in women’s sport for themselves and others like them. This includes but is not limited to women of color, queer women, women with disabilities, women representing religious minorities, and women from lower social classes (45, 53–57). The premise is that women’s sport typically favors women with privilege and power (45, 52, 59), so when women who lack these structural advantages participate, they are challenging the norms of women’s sport. As such, for example, when a woman athlete holds a marginalized racial or ethnic identity, everyday resistance within women’s sport is more likely to manifest (5, 6). In addition, given that some individuals may simultaneously hold both privileged and marginalized identities (4), it is important to understand how at times women athletes are resisting the norms of resistance and how at other times they are complicit in perpetuating oppressive norms against other women.

## Everyday resistance in future women’s sport research

Future research is needed to empirically examine this conceptual model. Following the four dimensions of everyday resistance of Johansson and Vinthagen (4), we suggest possible future lines of inquiry. First, to advance the understanding of sport as a repertoire, scholars could further explore the two levels of everyday resistance we identify in this model (women’s sport as everyday resistance and everyday resistance within women’s sport). For example, it is possible that different ways of participating (e.g., highly competitive, pick up, mixed gender) have varied implications for everyday resistance and women’s sport. Second, scholars could focus more specifically on each agent’s relationship to the levels we identify above. For example, how do sport’s powerbrokers understand women’s sport? Do they see one level of resistance as more threatening? How has their understanding of the symbolic and material meaning of women’s sport changed overtime? Further, what meaning do fans make of women’s sport [c.f. (1, 2, 59)]? How do they understand everyday resistance within women’s sport? Moreover, how do women athletes relate to these ideas? How often do they intentionally use women’s sport as everyday resistance or deliberately engage in everyday resistance within women’s sport? How does their mindset change over time or even perhaps day to day? Lastly, as spatialization and temporalization suggest that context plays a significant role in establishing how resistance manifests, future research could more specifically develop understandings of how these levels operate in specific sports, all of which represent unique sociocultural spaces.

We encourage scholars from all paradigmatic orientations to explore these research questions, however, qualitative methods may be best to understand the nuances of everyday resistance. Ethnographic research, for instance, may be most appropriate if scholars are interested in understanding how participants engage in mundane acts to resist power dynamics, or gendered norms, in mixed gender sport leagues [c.f. (6)]. In addition, semi-structured interviews can help scholars better understand the perspectives of women athletes about how they use sport to resist the norms of society and sport.

## Discussion

This paper helps advance scholarship on everyday resistance and scholarship that assesses resistance in sport. Scott (3) coined the term everyday resistance to draw attention to the ways that people with less power use tools at their disposal to incrementally advance cultural change. We conceptualize the role of sport in these efforts. In addition, as limited literature on everyday resistance has taken an intersectional approach (4), we extend the literature by explicitly exploring the layers of everyday resistance for women with multiple marginalized identities. That is, on the basis of their gender, these women engage in women’s sport as everyday resistance, while on the basis of their intersecting marginalized identities, they simultaneously engage in everyday resistance within women’s sport.

Our conceptual model follows in the tradition of past research that has explored resistance in and through sport [e.g. (10, 13, 18–21)]. However, as very limited sport research has embraced everyday resistance (3), we add to this emerging literature base (5–7). Specifically, by centering women’s gender and taking an intersectional approach (58), our conceptual model advances the understanding of *everyday resistance* in sport and lays the groundwork for others to further investigate how women’s sport is a form of everyday resistance.

We hope this conceptual model can be useful for scholars seeking to understand resistance in and through sport as well as the unique theoretical context of women’s sport. With respect to the former, using everyday resistance as an analytical lens can help sport scholars be precise in articulating who is resisting, what they are resisting, and how. Specifically, everyday resistance can help scholars conceptualize how at times sport activity itself is an act of resistance (5, 7). Second, conceptualizing the relationship between women’s sport and everyday resistance helps advance an understanding of the unique dynamics that women’s sport navigates. Developing an understanding of the unique space that women’s sport inhabits advances theory and can help explain why some sport management constructs operate differently in men’s and women’s sport [e.g., team identification (1), fan consumption patterns (60)].

There are limitations to our conceptual model and opportunities for future development. Given the necessity of situating resistance within specific relations of power, the model is primarily situated in US contexts. It would be fruitful to map a conceptual model that addresses women’s sport globally, or at the very least in other spatial and temporal norms. To further advance intersectional analyses, scholars should explicitly address additional axes of marginalization. Given our focus on women’s resistance, researchers should explore how athletes of other genders use sport as a tool of everyday resistance, and how the resistances of cis and trans athletes may differ given the contested terrain of inclusion (61). Future researchers should also consider everyday resistance through and in sport from the perspective of other stakeholders, including fans and those who hold structural power.

## Conclusion

This paper conceptualized a model for understanding everyday resistance and women’s sport. We first reviewed resistance, emphasizing everyday resistance, before identifying two levels of everyday resistance for women’s sport*:* women’s sport as everyday resistance and everyday resistance within women’s sport. In doing so, we introduced everyday resistance as a way to understand the revolutionary potential of women athletes, advancing both sport scholarship and everyday resistance literature. We hope scholars find this model useful as they seek to understand the way women create change in sport and in society—as well as how women athletes create change within women’s sport, specifically.

## References

[1] Delia EB. The psychological meaning of team among fans of women’s sport. J Sport Manage. (2020) 34(6):579–90. 10.1123/jsm.2019-0404

[2] Guest AM Luijten A. Fan culture and motivation in the context of successful women’s professional team sports: a mixed-methods case study of Portland Thorns fandom. Sport Soc. (2018) 21(7):1013–30. 10.1080/17430437.2017.1346620

[3] Scott JC. Weapons of the weak: Everyday forms of peasant resistance. New Haven: Yale University Press (1985).

[4] Johansson A Vinthagen S. Dimensions of everyday resistance: an analytical framework. Critic Sociol. (2016) 42(3):417–35. 10.1177/0896920514524604

[5] Klein A. Engaging acrimony: performing Lakota basketball in South Dakota. Soc Sport J. (2018) 35:58–65. 10.1123/ssj.2016-0177

[6] Mohamed AR. Black men on the blacktop: basketball and the politics of race. Boulder: Lynne Rienner Publishers (2017).

[7] Osmond G. Decolonizing dialogues: sport, resistance, and Australian aboriginal settlements. J Sport Hist. (2019) 46(2):288–301. 10.5406/jsporthistory.46.2.0288

[8] Hollander JA Einwohner RL. Conceptualizing resistance. Sociol Forum. (2004) 19(4):533–54. 10.1007/s11206-004-0694-5

[9] Kaufman P Wolff EA. Playing and protesting: sport as a vehicle for social change. J Sport Soc Issues. (2010) 34(2):154–75. 10.1177/0193723509360218

[10] Agyemang KJA Singer JN Weems AJ. ‘Agitate! agitate! agitate!’: sport as a site for political activism and social change. Organization. (2020) 27(6):952–68. 10.1177/1350508420928519

[11] Cooper JN Macaulay C Rodriguez SH. Race and resistance: a typology of African American sport activism. Sociol Sport. (2019) 54(2):151–81. 10.1177/1012690217718

[12] Numerato D. Between small everyday practices and glorious symbolic acts: sport-based resistance against the communist regime in Czechoslovakia. Sport Soc. (2010) 13(1):107–20. 10.1080/17430430903377920

[13] Carrington B. Sport, masculinity, and black cultural resistance. J Sport Soc Issues. (1998) 22(3):275–98. 10.1177/019372398022003004

[14] Dewar AM. Incorporation of resistance?: towards an analysis of women’s responses to sexual oppression in sport. Int Rev Sociol Sport. (1991) 26(1):15–23. 10.1177/101269029102600103

[15] Kukreja R. Using indigenous sport as resistance against migrant exclusion: Kabaddi and South Asian male migrants in Greece. J Ethn Migr Stud. (2023) 49(5):1173–90. 10.1080/1369183X.2022.2036954

[16] Beal B. Disqualifying the official: an exploration of social resistance through the subculture of skateboarding. Sociol Sport J. (1995) 12:252–67.

[17] Broad KL. The gendered unapologetic: queer resistance in women’s sport. Sociol Sport J. (2001) 18:181–204. 10.1123/ssj.18.2.181

[18] Carr JN. Skateboarding in dude space: the roles of space and sport in constructing gender among adult skateboarders. Sociol Sport J. (2017) 34:25–34. 10.1123/ssj.2016-0044

[19] Caudwell J. Sporting gender: women’s footballing bodies as sites/sights for the (re) articulation of sex, gender, and desire. Sociol Sport J. (2003) 20:371–86. 10.1123/ssj.20.4.371

[20] Davies SG Deckert A. Pretty strong women: ingenious agency, pink gloves and Muay Thai. Sociol Sport J. (2019) 36(3):213–23. 10.1123/ssj.2018-0145

[21] Ravel B Rail G. On the limits of “gaie” spaces: discursive constructions of women’s sport in Quebec. Sociol Sport J. (2007) 24:402–20. 10.1123/ssj.24.4.402

[22] Chase LF. (Un)disciplined bodies: A Foucauldian analysis of women’s rugby. Sociol Sport J. (2006) 23:229–47.

[23] Markula P. The technologies of the self: sport, feminism, and Foucault. Sociol Sport J. (2003) 20:87–107.

[24] Scott JC. Everyday forms of resistance. Copenhagen Papers. (1989) 4:33–62.

[25] Bayat A. From ‘dangerous classes’ to ‘quiet rebels’: politics of the urban subaltern in the Global South. Int Sociol. (2000) 15(3):533–57. 10.1177/026858000015003005

[26] Allison R. Kicking center: gender and the selling of women’s professional soccer. New Brunswick: Rutgers University Press (2018).

[27] Baxter K. What made the U.S. women’s World Cup win in 1999 such a pivotal moment? Los Angeles Times. (2019). Available at: https://www.latimes.com/sports/soccer/la-sp-united-states-1999-world-cup-team-20190603-story.html.

[28] Scott JC. Forward. In: Johansson A Vinthagen S, editor. Conceptualizing ‘everyday resistance’: a transdisciplinary approach. New York: Routledge (2020). p. ix–xi.

[29] Longman J. The girls of summer: the U.S. Women’s soccer team and how it changed the world. New York: HarperCollins (2001).

[30] Kliegman J. Nothing and everything has changed for the USWNT. The Ringer. (2019). Available at: https://www.theringer.com/soccer/2019/6/10/18656696/us-womens-national-team-world-cup-lawsuit-1999-megan-rapinoe.

[31] Metheny E. Symbolic forms of movement: the feminine image in sports. In: Metheny E, editors. Connotations of movement in sport and dance. Dubuque, IA: Brown (1965). p. 43–56.

[32] Fink JS Kane MJ LaVoi NM. The freedom to choose. Elite female athletes’ preferred representations within endorsement opportunities. J Sport Manag. (2014) 28:207–19. 10.1123/jsm.2013-0101

[33] Sartore-Baldwin ML. Sexual minorities in sports: prejudice at play. Boulder: Lynn Rienner (2013).

[34] Chalabaev A Sarrazin P Fontayne P Bioché J Clément-Guillotin C. The influence of sex stereotypes and gender roles on participation and performance in sport and exercise: review and future directions. Psychol Sport Exerc. (2013) 14:136–44. 10.1016/j.psychsport.2012.10.005

[35] Sullivan Barak K Kaunert CA Krane V Ross SR. Divergent perspectives: Post-Title IX sportkids’ views of female athletes. Women Sport Phys Act J. (2021) 29:152–63. 10.1123/wspaj.2021-0024

[36] Kunkel T Biscaia R Arai A Agyemang K. The role of self-brand connection on the relationship between athlete brand image and fan outcomes. J Sport Manag. (2020) 34(3):201–16.

[37] Cooky C Council LD Mears MA Messner MA. One and done: The long eclipse of women’s televised sports, 1989-2019. Commun. Sport. 9(3):347–71.

[38] Goggin G Newell C. Crippling paralympics?: media, disability, and Olympism. Media Int Aust. (2000) 97:71–83.

[39] Cavalier ES. Men at sport: gay men’s experiences in the sport workplace. J Homosexual. (2011) 58:626–46. 10.1080/00918369.2011.563662 21534074

[40] Luhn A. Women break an Olympic barrier, keep reproductive organs intact. Nation (2014). Available at: https://www.thenation.com/article/archive/women-break-olympic-barrier-keep-reproductive-organs-intact/.

[41] Crawford A. Justine Siegal, first woman to coach for an MLB team, is still breaking barriers for girls in baseball. ESPN (2019). Available at: https://www.espn.com/espnw/voices/story/_/id/27873456/justine-siegal-first-woman-coach-mlb-team-breaking-barriers-girls-baseball.

[42] Miller J. A. Finally honoring Bobbi Gibb, the first woman to run the Boston Marathon. ESPN (2016). Available at: https://www.espn.com/espnw/culture/feature/story/_/id/15190954/50-years-later-paying-tribute-bobbi-gibb-first-woman-run-boston-marathon.

[43] Boston Athletic Association. About us. Boston Athletic Association (n.d.). https://www.baa.org/about/about-us.

[44] Brown GS. A girl in a man’s game. Sports Illustrated. (1966). Available at: https://vault.si.com/vault/1966/05/02/a-game-girl-in-a-mans-game.

[45] Allison R. Privileging difference: negotiating gender essentialism in U. S. Women’s professional soccer. Sociol Sport J. (2020) 38(2):158–66. 10.1123/ssj.2020-0016

[46] Amdur N. (1984). Outlook is shiny and goals are set for Tiffany Chin. New York Times. (1984). Available at: https://www.nytimes.com/1984/02/13/sports/outlook-is-shiny-and-goals-are-set-for-tiffany-chin.html.

[47] Baer J. Changed the game: Before Kristi Yamaguchi and Michelle Kwan, there was Tiffany Chin. Yahoo (2021). Available at: https://www.yahoo.com/now/womens-history-tiffany-chin-asian-american-figure-skating-royalty-120045310.html.

[48] Yang J. Has U.S. figure skating at last given up on the ‘golden girl’? Huffington Post (2018). Available at: https://www.huffpost.com/entry/opinion-yang-figure-skating-racism_n_5a8ae67fe4b00bc49f46d853.

[49] Carter-Francique AR. ‘Re‘presenting ‘gabby’: examining the digital media coverage of Gabrielle Douglas at the 2012 London Olympic games. Int J Sport Stud. (2014) 4(9):1080–91.

[50] Kitchener C. Nia Dennis rejected ‘cookie cutter’ White gymnastics. Her Black Lives Matter-inspired routine is going viral. The Lilly (2021). Available at: https://www.thelily.com/nia-dennis-rejected-cookie-cutter-white-gymnastics-her-black-lives-matter-inspired-routine-is-going-viral.

[51] de la Cretaz B. Androgyny is now fashionable in the W.N.B.A. New York Times. (2019). Available at: https://www.nytimes.com/2019/06/18/opinion/androgyny-wnba-fashion.html.

[52] Isard RF Melton EN. Does sport media raise her name? Examining intersectional representation in media narratives. *Sport*. Bus Manage. (2021) 12(3):305–22. 10.1108/SBM-02-2021-0015

[53] Borland JF Bruening JE. Navigating barriers: a qualitative examination of the under-representation of black females as head coaches in collegiate basketball. Sport Manage Rev. (2010) 13:407–20. 10.1016/j.smr.2010.05.002

[54] Cunningham GB Hussain U. The case of LGBT diversity and inclusion in sport business. Sport Entertain Rev. (2020) 5(1).

[55] Fay T. Disability in sport: it’s our time: from the sidelines to the frontlines (title IX—b). J Intercolleg Sport. (2011) 4:63–94. 10.1123/jis.4.1.63

[56] Toffoletti K Palmer C. New approaches for studies of Muslim women and sport. Int Rev Sociol Sport. (2017) 52(2):146–63. 10.1177/1012690215589326

[57] Walker NA Melton EN. The tipping point: the intersection of race, gender, and sexual orientation in intercollegiate sports. J Sport Manage. (2015) 29:257–71. 10.1123/jsm.2013-0079

[58] Burton L. Underrepresentation of women in sport leadership: a review of research. Sport Manage Rev. (2015) 18(2):155–65. 10.1016/j.smr.2014.02.004

[59] Doyle JP Kunkel T Kelly SJ Filo K Cuskelly G. Seeing the same things differently: exploring the unique brand associations linked to women’s professional sport teams. J Strateg Market. (2021):1–15. 10.1080/0965254x.2021.1922489

[60] Katz M Melton EN Agha N Isard RF. Fan networks in women’s sport: An egocentric analysis of social fans and isofans. Sport Mark Q. (in press).

[61] Cunningham GB Isard R Melton EN. Transgender inclusion in sport. Kinesiol Rev. (2022) 11:64–70.

